# AlphaFold accelerates artificial intelligence powered drug discovery: efficient discovery of a novel CDK20 small molecule inhibitor†

**DOI:** 10.1039/d2sc05709c

**Published:** 2023-01-10

**Authors:** Feng Ren, Xiao Ding, Min Zheng, Mikhail Korzinkin, Xin Cai, Wei Zhu, Alexey Mantsyzov, Alex Aliper, Vladimir Aladinskiy, Zhongying Cao, Shanshan Kong, Xi Long, Bonnie Hei Man Liu, Yingtao Liu, Vladimir Naumov, Anastasia Shneyderman, Ivan V. Ozerov, Ju Wang, Frank W. Pun, Daniil A. Polykovskiy, Chong Sun, Michael Levitt, Alán Aspuru-Guzik, Alex Zhavoronkov

**Affiliations:** a Insilico Medicine Shanghai Ltd Suite 901, Tower C, Changtai Plaza, 2889 Jinke Road. Pudong New District Shanghai 201203 China alex@insilico.com; b Insilico Medicine Kong Kong Ltd Unit 310, 3/F, Building 8W, Phase 2, Hong Kong Science Park, Pak Shek Kok Hong Kong China; c Department of Chemistry, Department of Computer Science, University of Toronto, Vector Institute for Artificial Intelligence, Canadian Institute for Advanced Research Toronto Ontario Canada alan@aspuru.com; d Department of Structural Biology, Stanford University Palo Alto CA USA

## Abstract

The application of artificial intelligence (AI) has been considered a revolutionary change in drug discovery and development. In 2020, the AlphaFold computer program predicted protein structures for the whole human genome, which has been considered a remarkable breakthrough in both AI applications and structural biology. Despite the varying confidence levels, these predicted structures could still significantly contribute to structure-based drug design of novel targets, especially the ones with no or limited structural information. In this work, we successfully applied AlphaFold to our end-to-end AI-powered drug discovery engines, including a biocomputational platform PandaOmics and a generative chemistry platform Chemistry42. A novel hit molecule against a novel target without an experimental structure was identified, starting from target selection towards hit identification, in a cost- and time-efficient manner. PandaOmics provided the protein of interest for the treatment of hepatocellular carcinoma (HCC) and Chemistry42 generated the molecules based on the structure predicted by AlphaFold, and the selected molecules were synthesized and tested in biological assays. Through this approach, we identified a small molecule hit compound for cyclin-dependent kinase 20 (CDK20) with a binding constant Kd value of 9.2 ± 0.5 μM (*n* = 3) within 30 days from target selection and after only synthesizing 7 compounds. Based on the available data, a second round of AI-powered compound generation was conducted and through this, a more potent hit molecule, ISM042-2-048, was discovered with an average Kd value of 566.7 ± 256.2 nM (*n* = 3). Compound ISM042-2-048 also showed good CDK20 inhibitory activity with an IC_50_ value of 33.4 ± 22.6 nM (*n* = 3). In addition, ISM042-2-048 demonstrated selective anti-proliferation activity in an HCC cell line with CDK20 overexpression, Huh7, with an IC_50_ of 208.7 ± 3.3 nM, compared to a counter screen cell line HEK293 (IC_50_ = 1706.7 ± 670.0 nM). This work is the first demonstration of applying AlphaFold to the hit identification process in drug discovery.

## Abstract 2

A novel CDK20 small molecule inhibitor discovered by artificial intelligence based on an AlphaFold-predicted structure demonstrates the first application of AlphaFold in hit identification for efficient drug discovery.

## Graphical abstract

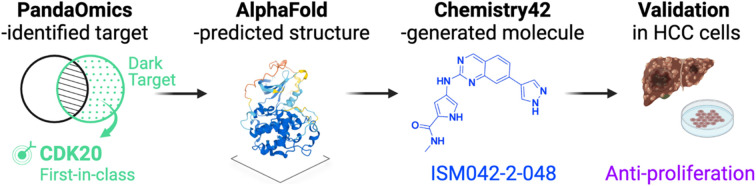

## Introduction

The 3D structures of proteins are highly correlated with their functions in cells and the biological impacts caused by amino acid mutations. A protein structure is a versatile tool to study the gene–disease association and mode of action (MoA), to evaluate the druggability of a therapeutic target. Structure-based drug discovery (SBDD) has been a mainstay method to identify hit molecules and perform lead optimization, which requires the 3D structure of a target.^2–4^ After an endeavor spanning decades, only a small fraction of known proteins have experimentally determined structures. Accurate protein structure prediction has been a longstanding challenge until the appearance of AlphaFold at CASP14.^5^ The structures predicted by AlphaFold can reach an accuracy level comparable to those of experimental methods.^6,7^ The scientific community celebrated DeepMind's accomplishment^8–12^ and the release of proteome-wide AlphaFold DB,^11^ which has now expanded to contain over 804 000 protein structures covering 21 species.^13^ Although the protein models predicted by AlphaFold have variable qualities from good, bad to ugly,^10^ the predicted local distance difference test score is provided as a confidence metric to guide the usage of 3D structures produced by AlphaFold. AlphaFold models have been used to aid the determination of experimental structures by crystallography^14^ and cryo-EM,^15^ to guide the functional study of PINK1,^16^ to help identify pathogenic mutations,^17,18^ and to explore the protein–protein interaction.^19^ Public databases include AlphaFold models as references, *e.g.*, UniProt,^20^ the therapeutic target database,^21^ and APPRIS.^22^ The methodology of AlphaFold has inspired RoseTTAFold,^23^ a potentially faster and cheaper protein prediction tool with adequate accuracy, and AlphaDesign,^24^ a protein design framework. AI-powered protein prediction has been selected as one of the 2021 breakthroughs by both Science^25^ and Nature^26^ journals.

In this work, we rapidly identify *de novo* molecules for a novel target by combining the protein structure predicted by AlphaFold with the end-to-end AI-powered drug discovery platforms PandaOmics and Chemistry42. The process embarked on indication, target selection, hit generation and hit identification.^27^ While we were aware of the capabilities of AlphaFold2 applied to the scientific community, the application and modification of the algorithm for commercial purposes are still poorly understood. Here, we used the freely-available predicted structures from the AlphaFold DB repository as a starting point.

**Fig. 1:**
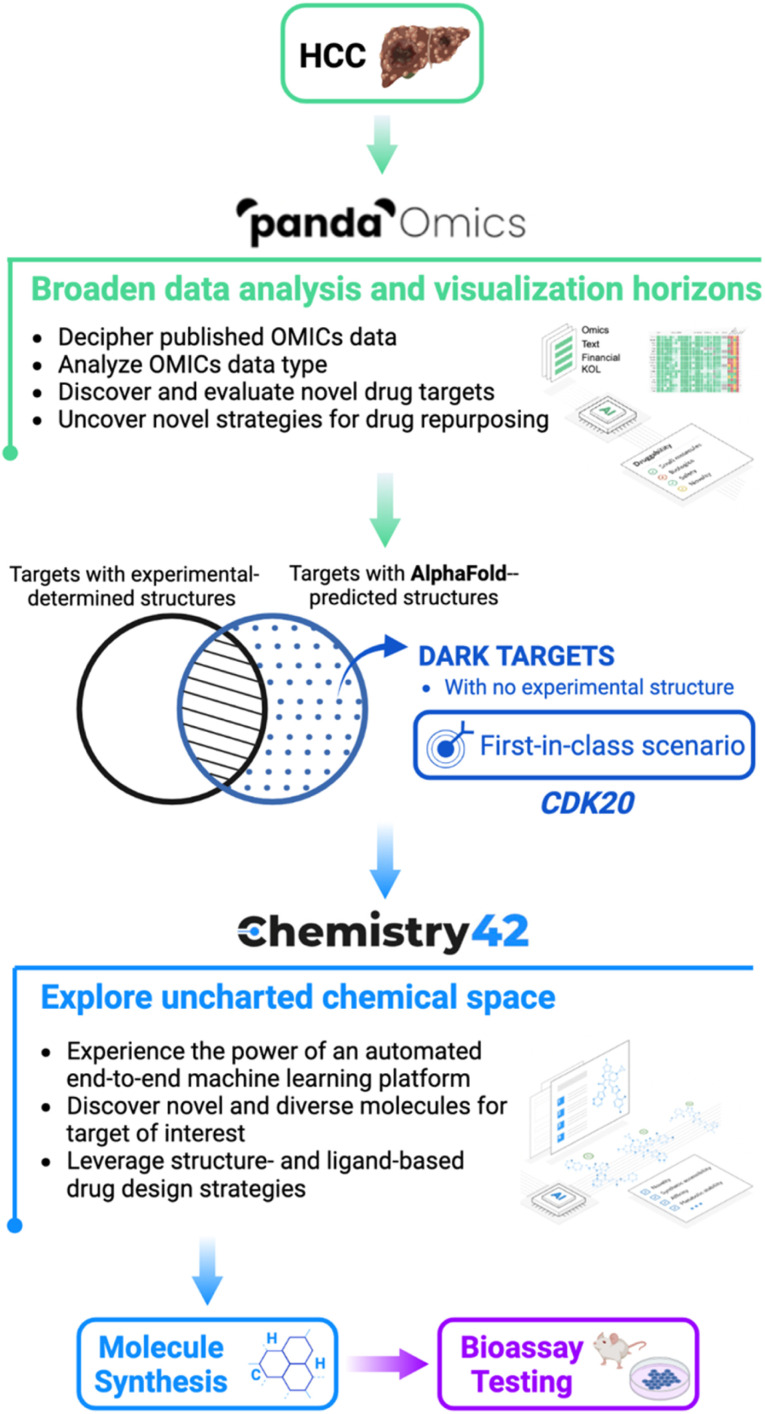
The pipeline to combine AlphaFold with Insilico Medicine end-to-end, and AI-powered drug discovery platforms PandaOmics and Chemistry42 in the drug discovery for hepatocellular carcinoma from target selection and hit generation to hit identification. A novel therapeutic target was identified from a pool of dark targets that have AlphaFold-predicted but lack experimentally determined structures. Such a target represents a first-in-class novel target which is revealed for the first time to treat HCC.

The general workflow is described in Fig. 1 where hepatocellular carcinoma (HCC) was used as the indication of interest due to its high prevalence in liver cancers and lack of effective treatments. In general, by analysis of text and OMICs data from 10 datasets for HCC, PandaOmics provided a list of the top 20 targets. Afterwards multidimensional filtration was applied including novelty, accessibility by biologics, safety, small molecule accessibility, and tissue specificity. Cyclin-dependent kinase 20 (CDK20) was finally selected as our initial target to work on due to its strong disease association, limited experimental structure information, and shortage of approved drugs or clinical compounds in the context of any disease during the last 3 years. Through Chemistry42 structure-based compound generation using the AlphaFold predicted CDK20 structure, 8918 molecules were generated and, after molecular docking and clustering, 7 were selected for synthesis and biological testing. Among them, compound ISM042-2-001 demonstrated a Kd value of 9.2 ± 0.5 μM (*n* = 3) in CDK20 kinase binding assay. Empowered by Chemistry42 and AlphaFold predicted protein structures, it took us only 30 days to discover our first hit. The predicted binding mode was then used as guidance for the second-round compound generation, synthesis, and testing, which resulted in a more potent hit molecule ISM042-2-048 with nanomolar potency. To the best of our knowledge, this work is the first reported example that successfully utilized AlphaFold-predicted protein structures to identify a confirmed hit for a novel target in early drug discovery.

## Target selection and identification

Primary liver cancer is the sixth most frequently occurring cancer and the third most common cause of worldwide cancer mortality according to the GLOBOCAN 2020 update released by the International Agency for Research on Cancer (IARC). Hepatocellular carcinoma (HCC) is the dominant type of liver cancer, accounting for approximately 75% of the total patient population. The incidence rate of liver cancer is very close to its mortality rate due to very poor prognosis in all regions around the world.^28^ PD-L1 inhibitor atezolizumab in combo with bevacizumab has become the new standard-of-care (SoC) first-line treatment for advanced HCC after demonstrating improvement in both 12 months overall survival (OS) and progression-free survival (PFS) compared to the previous SoC Nexavar,^29^ but there's still a huge unmet medical need for HCC patients.

PandaOmics is an automated drug discovery AI engine to accelerate and optimize key steps in the early stages of drug discovery.^30^ This biocomputational platform combines bioinformatics methods for data analysis, visualization and interpretation with advanced multimodal deep learning approaches for target identification.^31–35^ The PandaOmics therapeutic target and biomarker identification system is based on the combination of multiple scores derived from text and OMICs data associating genes with a disease of interest. Text evidence prioritization (text, financial and key opinion leader (KOL) score families) singles out the genes, which are extensively mentioned across the scientific literature and grant description. OMICs-based scores, in contrast, explore the molecular connection of genes with diseases based on differential expression, gene variants, interactome topology, signaling pathway perturbation analysis algorithms,^36^ knockout/overexpression experiments and more. This approach allows users to unveil hidden hypotheses that might not be obvious from common general knowledge or simple bioinformatics analysis. AI tools are extremely helpful for efficient target hypothesis generation. The overall scoring approach results in a ranked list of target hypotheses for a given disease which can be subsequently filtered according to their novelty, accessibility by small molecules and antibodies, safety, tissue specificity, crystal structure availability and major biological structures.^33,34^

Another unique feature of the PandaOmics platform is its ability to combine the data from different experiments into a single meta-analysis and leverage the insights from all the datasets together for precise target prioritization. We created a meta-analysis for each of the diseases of interest composed of 10 datasets for HCC (1133 disease samples and 674 healthy controls). After obtaining the ranked list of target hypotheses we applied PandaOmics filters in order to get a list of the most promising targets that satisfy the first-in-class scenario (see the Methods section) characteristics and share the current unavailability of crystal structures but have structure folds predicted by AlphaFold. The final list of top 20 targets was then manually curated as the most promising candidates. For the HCC case, CDK20 was chosen due to its highest scores aligned with the first-in-class scenario. The proposed therapeutic target CDK20 was passed to the Chemistry42 platform for the automated generation of small molecule inhibitors.

## CDK20 as a promising target for cancer treatment

CDK20, also known as cell cycle-related kinase (CCRK), is the latest identified member of the cyclin-dependent kinase family, which has attracted great attention in recent years due to its functions (both cell cycle-dependent and -independent) in a variety of human tissues.^37^ CDK20 is widely expressed at a comparable translational level in many human tissues including the brain, lung, liver, pancreas, and gastrointestinal tract.^38^ More importantly, increasing preclinical evidence suggested that CDK20 is overexpressed in many tumor cell lines including tumor samples from patients with different types of cancer, such as colorectal cancer, hepatocellular carcinoma (HCC), lung cancer, and ovarian carcinoma.^39–42^*In vitro* studies showed that the androgen receptor (AR), CDK20, and β-catenin constitute a positive feedback circuit to promote cell cycle progression in HCC cells, and CDK20 overexpression frequently correlates with ectopic expression of the AR and β-catenin in primary HCC tissue samples and with tumor staging and poor overall survival of patients.^40^ In lung cancer cells, CDK20 competes with nuclear factor erythroid 2-related factor 2 (NRF2) for kelch-like ECH associated protein 1 (KEAP1) binding, which prevents degradation of NRF2 and enhances its transcriptional activity, therefore lowering the cellular reactive oxygen species (ROS) level. Moreover, CDK20 depletion in lung cancer cells demonstrates impaired cell proliferation, defective G2/M arrest, and increased radiochemosensitivity.^41^ In addition to its pro-tumorigenic role through modulation of the cell cycle and oncogenic signaling, CDK20 is also involved in immunosuppression in certain types of tumors. Zhou *et al.* reported that by activating the EZH2-NF-κB pathway, CDK20 expressed in HCC cells increased IL-6 production and induced immunosuppressive MDSC expansion from human peripheral blood mononuclear cells; inhibition of tumorous CDK20 increased IFN-γ + TNF-α + CD8^+^ T cell infiltration and upregulated PD-L1 expression level in tumors, providing a greater chance of combination therapy with PD-L1 blockade to eradicate HCC tumors.^43^ Hence emerging scientific evidence suggested that CDK20 inhibition could be considered a promising therapeutic approach for cancer treatment, especially for HCC.

## Generation of novel hits targeting CDK20 by using AlphaFold predicted structures

**Fig. 2:**
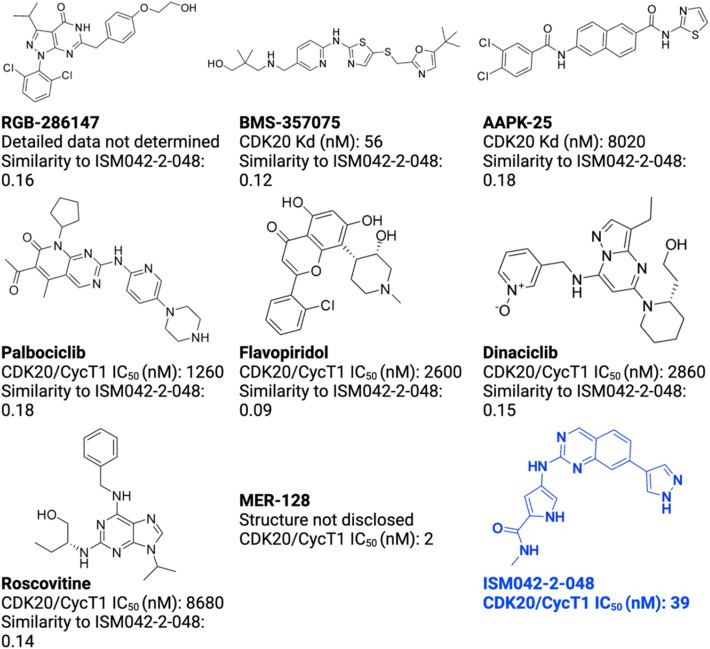
Reported CDK20 inhibitors from the literature and the novel inhibitor ISM042-2-048 discovered in this paper. The Tanimoto similarities of reported molecules to ISM042-2-048 are calculated from Morgan fingerprints using RDKit.^1^

**Fig. 3:**
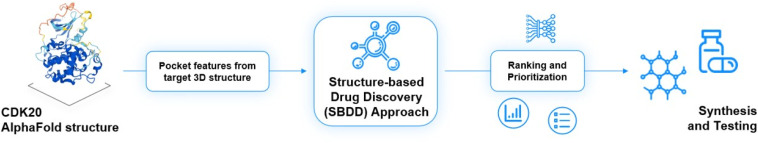
Insilico Medicine generative procedures for CDK20 hits.

As of today, there are limited CDK20 inhibitors reported (displayed in Fig. 2) despite great success being achieved with inhibitors against other members of the CDK family. One possible reason is that there is no available 3D-structure information for this target. In 2005, Nikolai and co-workers described a potential CDK20 inhibitor named RGB-286147 without reporting any binding affinity data.^44^ Eurofins disclosed BMS-357075 as a CDK20 binder with a Kd value of 56 nM.^45^ AAPK-25 was also reported as a CDK20 inhibitor with a Kd value of 8020 nM.^46^ Moreover, Mueller *et al.* described several potential CDK20 inhibitors in their recent poster, including Palbociclib, Flavopiridol, Dinaciclib, and Roscovitine with IC_50_ values ranging from 1260 nM to 8680 nM.^47^ Besides, they also identified another potent CDK20 inhibitor MER-128 with an IC_50_ value of 2 nM; however, the structure of this molecule was not disclosed.^47^Fig. 3 describes our generative procedures for the identification of CDK20 inhibitors starting from structure extraction to hit generation through the SBDD approach by utilizing Chemistry42.^27,48–55^

After uploading a protein structure to the Chemistry42 platform, the built-in energy-based approach is automatically used in order to determine putative binding sites. The surface of the protein is evenly covered with probes (methyl), and the energy of non-covalent interactions with the receptor atoms is calculated for each probe. Probes having energy lower than a user-defined threshold are clustered into separate pockets. Each identified cavity is scored using pocket volume, surface, and depth descriptors. Based on these descriptors, Chemistry42 provides a list of identified binding sites.

**Fig. 4:**
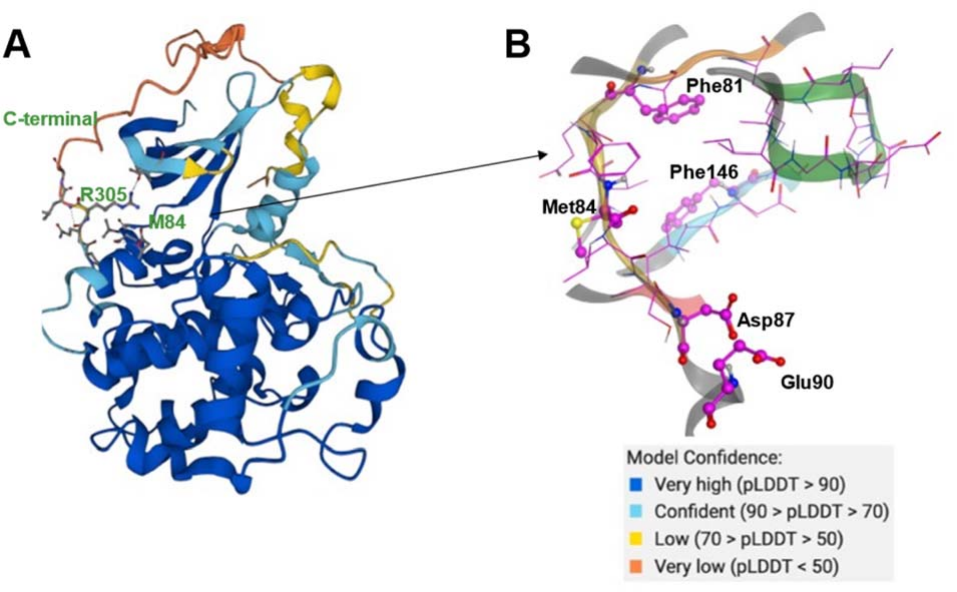
(A) The AlphaFold predicted structure of CDK20 (AF-Q8IZL9-F1-model_v1); (B) ATP pocket of CDK20 with a DFG-in (residue Phe146) conformation. Met84 is the hinge residue. P-loop is colored in green. Two acid centers Asp87 and Glu90 are located in the solvent-exposed region of the protein.

The CDK20 structure predicted by AlphaFold (AF-Q8IZL9-F1-model_v1) has a high confidence level overall except for the C-terminal as displayed in Fig. 4A. The C-terminal conformation in the AlphaFold predicted structure with very low confidence blocks the solvent-exposed region of the protein and the residue Arg305 on the C-terminal occupies the ATP pocket as shown in Fig. 4A. The C-terminal has a flexible loop that has various conformations. The C-terminal in the AlphaFold predicted structure is not in a favorable conformation for the design of an inhibitor by occupying the ATP pocket. Hence the C-terminal (Pro303-Gly346) is removed and only the structure from residue Met1 to residue Ile302 is used as an input for molecule generation in Chemistry42. For this structural model, Chemistry42 identified a shallow ATP binding pocket with an estimated volume of around 150 Å^3^ as shown in Fig. 4B. Near the hinge residue Met84, residue Phe81 occupies the gatekeeper and stops a ligand from reaching the back pocket. The predicted binding pocket has a DFG-in conformation and two acidic centers (Asp87 and Glu90) in the solvation region. A pocket-based generation approach was then utilized to generate novel molecule structures. The hinge residue Met84 is defined as the required binding point. Other 3D structural information from the ATP pocket has been used to guide the generation of molecules towards better fitting the targeted pocket, such as the 3D shape of the pocket, the pocket volume, and the spatial arrangements of atoms in the pocket. In total 8918 molecules were designed by Chemistry42. After molecular docking and clustering, 54 molecules with diverse hinge core structures were prioritized and 7 compounds were selected for synthesis.

## Results and discussion

**Fig. 5:**
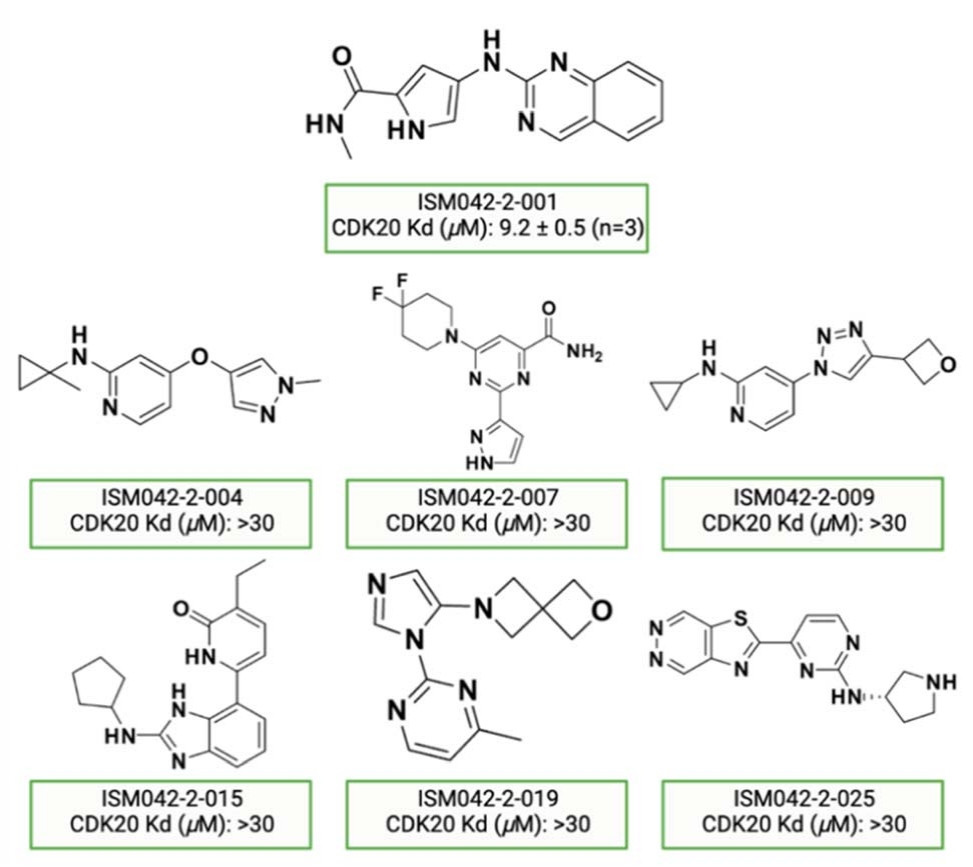
Chemical structures for the selected 7 molecules from the first-round Chemistry42 generation for synthesis and testing in CDK20 binding assay.

**Fig. 6:**
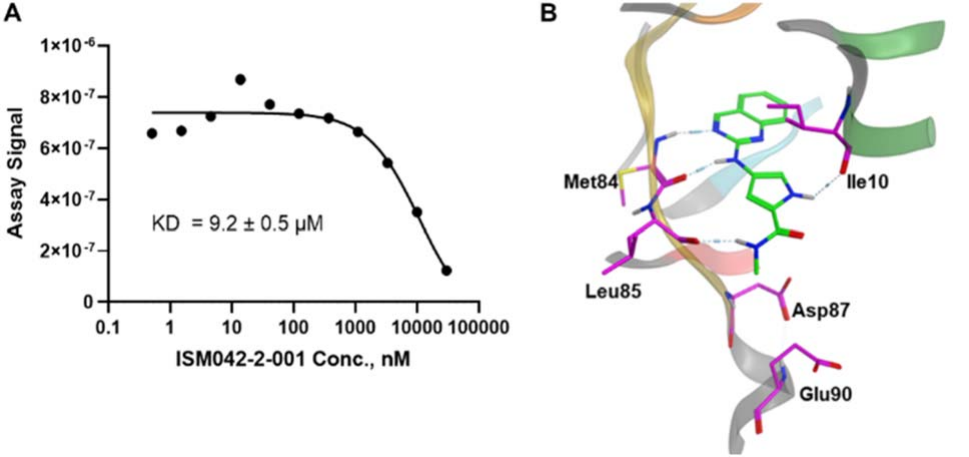
(A) Representative binding affinity curve of ISM042-2-001 in CDK20 kinase binding assay. Data points are presented as the mean of duplicate wells in one experiment. Similar results were obtained in three independent experiments and the KD is the mean ± SD of three independent experiments. (B) Predicted binding pose for ISM042-2-001 with CDK20.

Fig. 5 shows the chemical structures for the 7 compounds selected to synthesize and to assess the binding abilities towards CDK20. Among the selected compounds, one compound ISM042-2-001 demonstrated a Kd value of 9.2 ± 0.5 μM (*n* = 3, one representative binding curve is shown in Fig. 6A) in CDK20 kinase binding assay and an IC_50_ value of >6000 nM in CDK20 kinase activity assay. It took us only 30 days to discover the hit molecule. We also proposed the binding mode for ISM042-2-001 *via* molecular docking as depicted in Fig. 6B: the four hydrogen bond interactions are represented as dashed lines. Besides the two hydrogen bonds formed with the hinge residue Met84, ISM042-2-001 also interacts with residue Leu85 *via* the amide-NH group and with residue Ile10 in the P-loop *via* the pyrrole-NH group. Alternatively, the amide-NH group or the pyrrole-NH group may form hydrogen bonds with the two acid centers Asp87 and Glu90 in the solvation region.

**Fig. 7:**
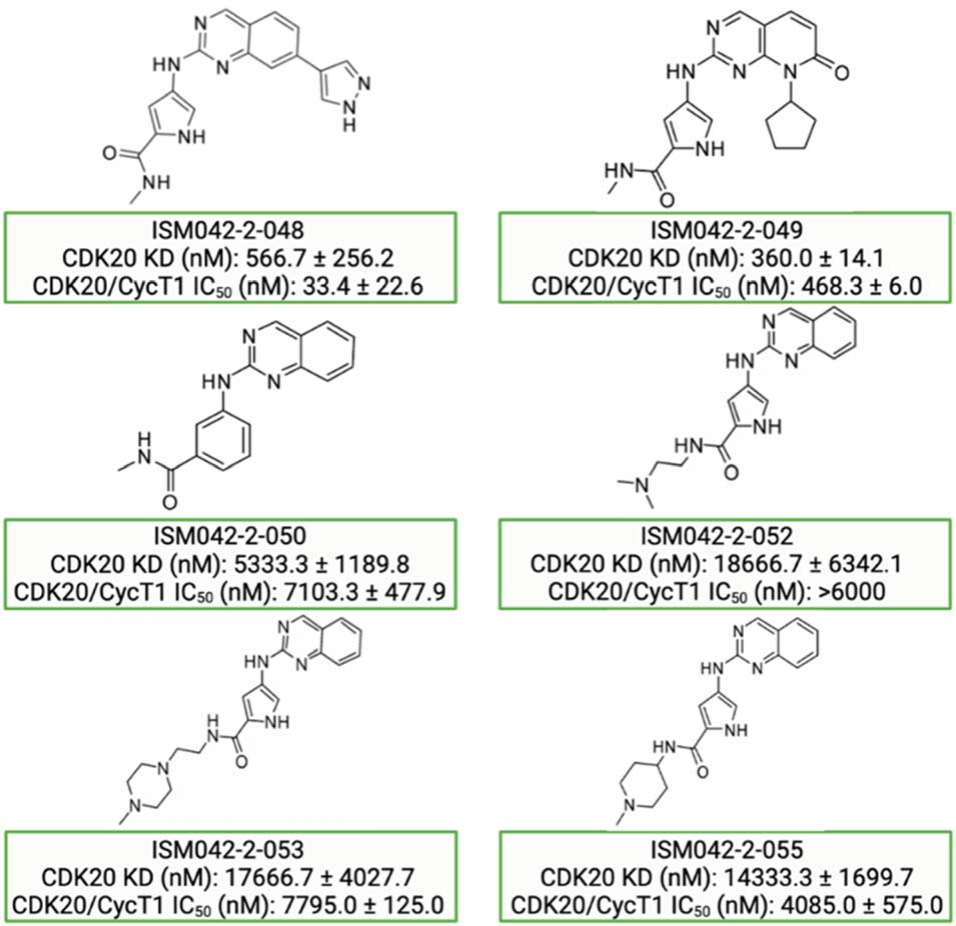
Chemical structures for the second-round Chemistry42 generation for synthesis and testing in CDK20 binding and kinase activity assays. Biological data with a standard deviation are presented from three independent experiments.

**Fig. 8:**
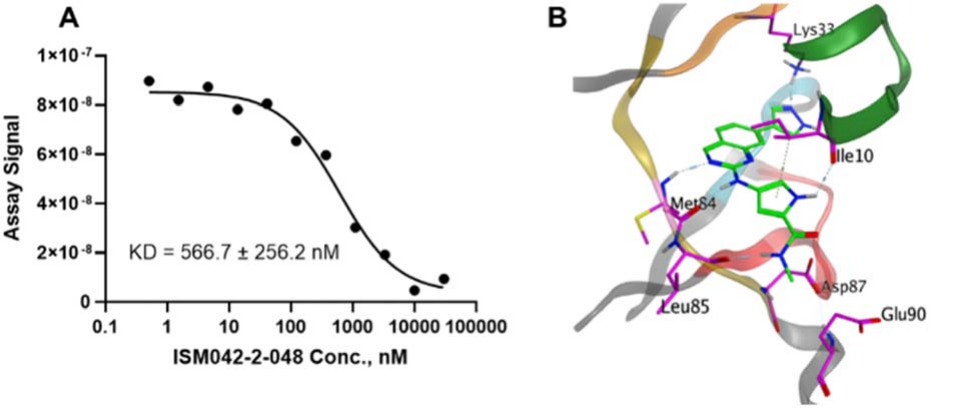
(A) Representative binding affinity curve for ISM042-2-048 in CDK20 kinase binding assay. Data points are presented as the mean of duplicate wells in one experiment. Similar results were obtained in three independent experiments and the KD is the mean ± SD of the three independent experiments. (B) Predicted binding pose for ISM042-2-048 in CDK20.

**Fig. 9:**
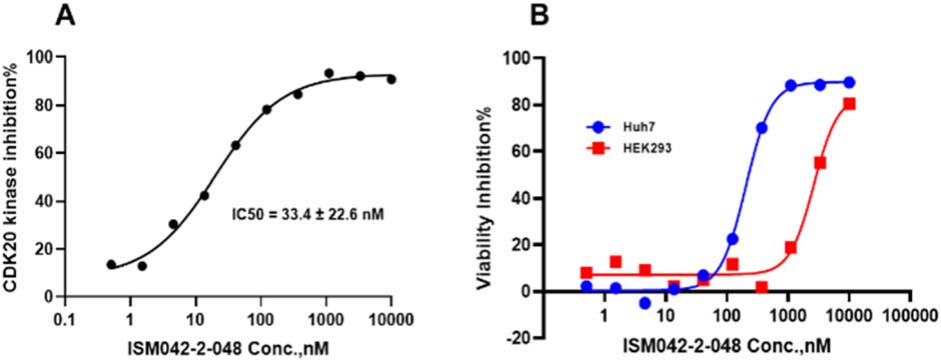
(A) Representative dose–response curve for ISM042-2-048 in CDK20 kinase activity assay. Similar results were obtained in three independent experiments and the IC50 is the mean ± SD of the three independent experiments. (B) Cell viability curves for ISM042-2-048 in cell line Huh7 and counter screen cell line HEK293. Data points are presented as the mean of duplicate wells in one experiment. Similar results were obtained in three independent experiments.

Based on the predicted binding pose and potency data, we conducted a second round of compound generation utilizing our generative AI tool Chemistry42. 16 novel molecules were generated aiming to improve the binding affinity based on two approaches: (1) occupation of the hydrophobic pocket near the gatekeeper region with functional groups on the quinazoline ring; (2) modifications of the pyrrole-2-carboxamide group to access the solvation region and interact with acidic residues Asp87 or Glu90. With the above strategies, 6 out of 16 generated molecules were synthesized and tested as shown in Fig. 7, of which ISM042-2-048 and ISM042-2-049 displayed 15 and 24 fold improvements in binding affinity compared to ISM042-2-001, with measured Kd values of 566.7 ± 256.2 nM and 360.0 ± 14.1 nM, respectively. A predicted binding mode of ISM042-2-048 with CDK20 was shown in Fig. 8B. Based on the proposed binding mode, in addition to the interactions in the hinge and solvent areas, the pyrazole group of ISM042-2-048 forms a hydrogen bond with residue Lys33, which explains the significant improvement of its binding affinity. ISM042-2-048 is different from the reported CDK20 inhibitors with a novel scaffold and low molecular similarity as shown in Fig. 2. Furthermore, the inhibition of CDK20 kinase activity by ISM042-2-048 was confirmed with an average IC_50_ of 33.4 ± 22.6 nM (*n* = 3) and showed selective anti-proliferation activity in Huh7 (IC_50_ = 208.7 ± 3.3 nM), an HCC cell line with CDK20 overexpression, compared to a counter screen cell line HEK293 (IC_50_ = 1706.7 ± 670.0 nM), as displayed in Fig. 9. Next-round of optimization will be initiated soon to further improve potency, and the ADME properties and kinase selectivity will also be evaluated.

## Conclusions

Structure-based drug discovery (SBDD) has been a mainstay method to identify hit molecules and perform lead optimization. And the predicted protein structure by AlphaFold has been considered a powerful tool to identify hits for novel targets with no or limited structure information. Herein, we present an example of rapid identification of a CDK20 hit molecule by using AlphaFold predictions as inputs to our automated drug discovery AI engines PandaOmics and Chemistry42 within 30 days covering target selection, molecule generation, compound synthesis and biological testing. Among the 7 compounds synthesized, ISM042-2-001 demonstrated a Kd value of 9.2 ± 0.5 μM (*n* = 3) in CDK20 kinase binding assay. Based on the preliminary SAR, a second round of AI-powered compound generation was conducted and 6 more compounds were synthesized and tested within 30 days from the discovery of the first hit ISM042-2-001. To our delight, a more potent hit molecule, ISM042-2-048, was discovered with an average Kd value of 566.7 ± 256.2 nM (*n* = 3) and an average IC_50_ value of 33.4 ± 22.6 nM (*n* = 3). Furthermore, ISM042-2-048 also demonstrated good anti-proliferation activity in an HCC cell line Huh7 with high expression levels of CDK20 (IC_50_ = 208.7 ± 3.3 nM) whereas less effect was seen in the counter screen cell line HEK293 (IC_50_ = 1706.7 ± 670.0 nM). This preliminary result indicated that our CDK20 inhibitor didn't induce indiscriminate cyto-toxicity but rather showed a stronger effect on CDK20-overexpressing HCC cells and therefore could serve as a tool molecule to evaluate biological functions for this target. Further optimization of this molecule as well as the evaluation of ADME properties are in progress. Moreover, this work represents the first example of successfully utilizing AlphaFold predicted protein structures for hit identification for a novel target. Further applications of this approach to other target classes such as GPCR and E3 ligase are ongoing.

## Materials and methods

### Target ID and target proposal

The PandaOmics platform was used to conduct hypothesis generation for hepatocellular carcinoma, limiting the target list to the proteins whose structures were predicted by AlphaFold2. HCC analysis combined the data from ten experiments: GSE36376,^56^ GSE107170,^56,57^ GSE102079,^58^ GSE45267,^58,59^ GSE133039,^60^ GSE104766,^61^ GSE77314,^61,62^ GSE60502,^63^ E-MTAB-5905 ^64^ and TCGA-LIHC,^65^ resulting in 1133 disease samples and 674 healthy controls. Targets are proposed by following our first-in-class scenario. The first-in-class scenario is defined as follows: the protein is druggable by small molecules, the target is considered novel by the PandaOmics system, and the target was not in phase 1 clinical trials in the context of any disease during the last 3 years and is not a target of previously approved drugs.

### CDK20 human CMGC kinase binding assay

The assay was available as a product of the KINOMEscan service from DiscoverX/Eurofins. In brief, CDK20 proteins were produced in HEK-293 cells and subsequently tagged with DNA for qPCR detection. Streptavidin-coated magnetic beads were treated with biotinylated small molecule ligands for 30 minutes at room temperature to generate affinity resins. The liganded beads were blocked with excess biotin and washed with blocking buffer (SeaBlock (Pierce), 1% BSA, 0.05% Tween 20, and 1 mM DTT) to remove the unbound ligand and to reduce non-specific binding. Binding reactions were assembled by combining kinases, liganded affinity beads, and test compounds in 1× binding buffer (20% SeaBlock, 0.17× PBS, 0.05% Tween 20, and 6 mM DTT). Test compounds were prepared as 111X stocks in 100% DMSO. Binding constants (Kds) were determined using an 11-point 3-fold compound dilution series with three DMSO control points. All compounds for Kd measurements are distributed by acoustic transfer (non-contact dispensing) in 100% DMSO. The compounds were then diluted directly into the assays such that the final concentration of DMSO was 0.9%. All reactions were performed in polypropylene 384-well plates. Each has a final volume of 0.02 ml. The assay plates were incubated at room temperature with shaking for 1 hour and the affinity beads were washed with washing buffer (1× PBS and 0.05% Tween 20). The beads were then re-suspended in elution buffer (1× PBS, 0.05% Tween 20, and a 0.5 μM non-biotinylated affinity ligand) and incubated at room temperature under shaking for 30 minutes. The kinase concentration in the eluates was measured by qPCR. Kds were calculated with a standard dose–response curve. The curves were fitted using a non-linear least squares fit with the Levenberg–Marquardt algorithm.

### CDK20 kinase activity assay

A radiometric protein kinase assay (^33^PanQinase^®^ ActivityAssay, available as a service product from Reaction Biology Corp.) was used for measuring the kinase activity of the CDK20 kinases. This assay was performed in 96-well FlashPlates™ from PerkinElmer (Boston, MA, USA) in a 50 μl reaction volume. The reaction cocktail was pipetted in four steps in the following order: (a) 25 μl of assay buffer (standard buffer/[γ-^33^P]-ATP); (b) 10 μl of ATP solution (in H_2_O); (c) 5 μl of test compound (in 10% DMSO); (d) 10 μl of enzyme/substrate mixture. The assay for CDK20 kinase contained 70 mM HEPES-NaOH pH 7.5, 3 mM MgCl_2_, 3 mM MnCl_2_, 3 μM Na-orthovanadate, 1.2 mM DTT, 50 μg ml^−1^ PEG_20000_, 1.0 μM ATP [γ-^33^P],-ATP (approx. 7 × 10^5^ cpm per well), and 200 ng/50 μl kinase protein, and the substrate was 4.0 μg/50 μl. The compounds were dissolved to 1 × 10^−3^ M in volumes of 100% DMSO. The 1 × 10^−3^ M stock solutions were subjected to a serial, semi-logarithmic dilution using 100% DMSO as a solvent. The final volume of the assay was 50 μL All compounds were tested at 10 final assay concentrations in the range from 1 × 10^−5^ M to 3 × 10^−10^ M. The final DMSO concentration in the reaction cocktails was 1% in all cases. The “low control” was defined as the value that reflects unspecific binding of radioactivity to the plate in the absence of a protein kinase but in the presence of the substrate. The “high control” was defined as the full activity in the absence of any inhibitor. The difference between high and low controls was taken as 100% activity. As part of the data evaluation the low control value from a particular plate was subtracted from the high control value as well as from all 80 “compound values” of the corresponding plate.

The residual activities for each concentration and the compound’s IC50 values were calculated using Quattro Workflow V3.1.1 (Quattro Research GmbH, Munich, Germany; http://www.quattro-research.com/). The fitting model for the IC50 determinations was “sigmoidal response (variable slope)” with parameters “top” fixed at 100% and “bottom” at 0%. The fitting method used was a least-squares fit.

### Cell viability assay

The Huh7 and HEK293 cells were maintained in DMEM with 10% FBS and 1% streptomycin and penicillin in 5% CO_2_. When the confluence of these two cell lines reached 80–90%, cells were harvested and resuspended, and the proper cells were added into a 384-well plate as below: Huh-7: 650 cells per well; HEK-293 : 300 cells per well. The final testing concentrations of the compounds were: 10 000, 3333, 1111, 370, 123, 41.2, 13.7, 4.57, 1.52, and 0.51 nM. The cells were incubated in a 37 °C, 5% CO_2_ incubator for 3 days, and then a reagent was added (Celltiter Glo assay kit) to the cells for testing and a Multiplate reader was used to record the chemiluminescence value. And GraphPad Prism 9 software was used to calculate IC50 and plot the effect–dose curve of compounds. The assays were conducted at Pharmaron Inc. in a fee-for-service mode.

## Code availability

Two products were used in this study: PandaOmics and Chemistry42. These platforms are commercially available online at https://www.pandaomics.com/ and https://www.chemistry42.com/ correspondingly. Several algorithms used on the platform are publicly available, including the IPANDA algorithm used in PandaOmics (https://github.com/insilicomedicine/ipanda), GENTRL model used as part of Chemistry42 (https://github.com/insilicomedicine/GENTRL), and VAE-TRIP used in Chemistry42 (https://github.com/insilicomedicine/TRIP). Demo of both platforms can be requested on the platforms under the “request demo” link.

## Data availability

AlphaFold structures are available online at https://alphafold.ebi.ac.uk/. Gene series data are publicly available at the GEO database (https://www.ncbi.nlm.nih.gov/geo/), ArrayExpress (https://www.ebi.ac.uk/arrayexpress/), and the Cancer Genome Atlas (TCGA, https://www.cancer.gov/about-nci/organization/ccg/research/structural-genomics/tcga).

## Author contributions

F. R. led the project. X. D. conducted compound selection and synthesis and drafted the initial version of the manuscript. M. Z. performed compound generation and docking. M. K. performed target identification. X. C. conducted biology and assay development and execution. W. Z. performed compound selection and synthesis. A. M. provided support to Chemistry42 for compound generation. A. A. developed Chemistry42 for compound generation. V. A. generated compounds from Chemistry42. Z. C. developed and executed assays. S. K. performed target selection and identification. X. L. and B. H. M. L. drew figures. Y. L. conducted compound docking. V. N., A. S. and I. V. O. performed target selection and identification. J. W. performed target identification and developed the assays. F. W. P. drew figures. D. A. P. provided support to Chemistry42 for compound generation. C. S. wrote, reviewed and edited the manuscript. M. L. and A. A.-G. conceived the project. A. Z. conceived and drove the project.

## Conflicts of interest

Insilico Medicine is a company developing an AI-based end-to-end integrated pipeline for drug discovery and development and engaged in aging and cancer research. Alán Aspuru-Guzik is co-founder and Chief Vision officer of Kebotix, an AI-powered materials and molecular discovery company and co-founder and Chief Scientific Officer of Zapata Computing, a quantum software computing company. Alán Aspuru-Guzik is a scientific advisor to Insilico Medicine. Michael Levitt is an advisor to Insilico Medicine.

## Supplementary Material

SC-014-D2SC05709C-s001
