# Supplementary material for: AlphaFold accelerates artificial intelligence powered drug discovery: efficient discovery of a novel CDK20 small molecule inhibitor
Source: Chem Sci. 2023 Jan 10;14(6):1443–52. doi: 10.1039/d2sc05709c (PMC9906638; doi:10.1039/d2sc05709c)
Supplement: SC-014-D2SC05709C-s001 [file SC-014-D2SC05709C-s001.pdf]

Electronic Supplementary Material (ESI) for Chemical Science.
This journal is © The Royal Society of Chemistry 2023

Electronic Supplementary Material (ESI) for Chemical Science.
This journal is © The Royal Society of Chemistry 2022

Supporting Information

AlphaFold Accelerates Artificial Intelligence Powered Drug Discovery: Efficient Discovery of a Novel CDK20 Small Molecule Inhibitor

Feng Ren[a], Xiao Ding[a], Min Zheng[a], Mikhail Korzinkin[b], Xin Cai[a], Wei Zhu[a], Alexey Mantsyzov[b], Alex Aliper[b], Vladimir Aladinskiy[b], Zhongying Cao[a], Shanshan Kong[a], Xi Long[b], Bonnie Hei Man Liu[b], Yingtao Liu[a], Vladimir Naumov[b], Anastasia Shneyderman[b], Ivan V. Ozerov[b], Ju Wang[a], Frank W. Pun[b], Daniil A. Polykovskiy[b], Chong Sun[c], Michael Levitt[d], Alán Aspuru-GuzikiD[c,*], and Alex ZhavoronkoviD[a,b,*]

[a] Insilico Medicine Shanghai Ltd, Suite 901, Tower C, Changtai Plaza, 2889 Jinke Road. Pudong New District,Shanghai 201203, China.

[b] Insilico Medicine Kong Kong Ltd, 307A, Core Building 1, 1 Science Park East Avenue, Hong Kong Science Park, Pak Shek Kok, Hong Kong.

[c] Department of Chemistry, Department of Computer Science, University of Toronto, Toronto, Ontario, Canada; Vector Institute for Artificial Intelligence, Toronto, Ontario, Canada; Canadian Institute for Advanced Research, Toronto, Ontario, Canada.

[d] Department of Structural Biology, Stanford University, Palo Alto, CA, USA.

* Corresponding authors: Alex Zhavoronkov, email: alex@insilico.com; Alán Aspuru-Guzik, email: alan@aspuru.com

Synthesis of compounds

ISM042-2-001

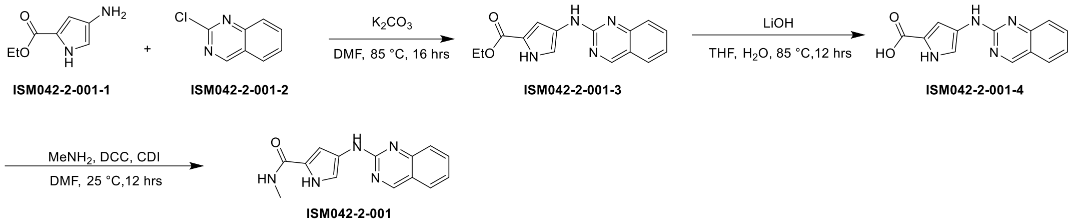

To a solution of **ISM042-2-001-1** (500 mg, 3.24 mmol, 1.0 equiv.) in DMF (15 mL) were added K_2CO_3 (672 mg, 4.86 mmol, 1.5 equiv.) and **ISM042-2-001-2** (534 mg, 3.24 mmol, 1.0 equiv.). The mixture was stirred at 80 °C under N_2 atmosphere for 16 hrs. The reaction mixture was diluted with water (30 mL) and extracted with EtOAc (15 mL × 3). The combined organic layers were washed with brine (30 mL × 2), dried over anhydrous sodium sulfate, filtered and concentrated. The crude was purified by silico gel chromatography (PE/EtOAc = 5/1) to afford **ISM042-2-001-3** (632 mg, 69.03% yield) as a yellow solid.

To a solution of **ISM042-2-001-3** (100 mg, 354 μmol, 1.0 equiv.) in THF (2 mL) was added LiOH (aq., 1 M, 1.77 mL, 5.0 equiv.). The mixture was stirred at 50 °C for 2 hrs. The reaction mixture was neutralized by HCl (aq.,1 M) and then extracted with EtOAc (3 mL × 3). The combined organic layers were washed with brine (5 mL × 2), dried over anhydrous sodium sulfate, filtered and concentrated to afford **ISM042-2-001-4** (120 mg, crude) as yellow oil. ^{1}H NMR (400 MHz, DMSO-d_6) δ 12.14 – 11.97 (m, 2H), 11.43 (br s, 1H), 9.67 (s, 1H), 9.19 (s, 1H), 7.84 (d, J = 8.00 Hz, 1H), 7.78 - 7.72 (m, 1H), 7.58 (br d, J = 8.40 Hz, 2H), 7.34 - 7.26 (m, 1H), 6.87 (s, 1H). LCMS [M-H]$^-$**:** 253.0.

To a solution of **ISM042-2-001-4** (100 mg, 393 μmol, 1.0 equiv.) and CH_3NH_2 (2 M, 1.97 mL, 10.0 equiv.) in DMF (0.2 mL) were added CDI (77 mg, 472 μmol, 1.2 equiv.) and DCC (122 mg, 590 μmol, 1.5 equiv.). The mixture was stirred at r.t. for 12 hrs. The reaction mixture was diluted with water (2 mL) and extracted with EtOAc (3 mL × 3). The combined organic layers were washed with brine (5 mL × 2), dried over anhydrous sodium sulfate, filtered and concentrated. The obtained residue was purified by prep-HPLC to afford **ISM042-2-001** (35.0 mg, 31.7% yield) as a yellow solid. ^{1}H NMR (400 MHz, $CDCl_3$) δ 9.24 - 9.02 (m, 2H), 7.85 - 7.69 (m, 3H), 7.57 - 7.47 (m, 1H), 7.43 - 7.33 (m, 1H), 6.68 (br s, 1H), 5.94 - 5.79 (m, 1H), 3.01 (d, J = 4.80 Hz, 3H). LCMS [M+H]$^+$: 268.0.

ISM042-2-004

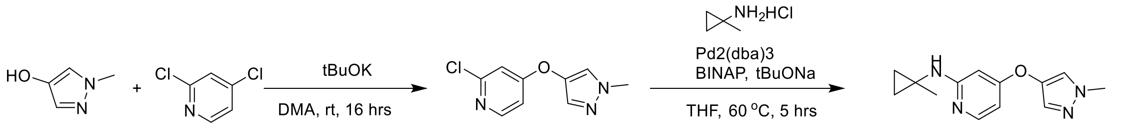

ISM042-2-004-1 ISM042-2-004-2 ISM042-2-004-3 ISM042-2-004

To a solution of **ISM042-2-004-1** (331 mg, 3.38 mmol, 1.0 equiv.) in DMA (10 mL) was added *t*BuOK (455 mg, 4.05 mmol, 1.2 equiv.). The mixture was stirred at r.t. for 1 hr, followed by the addtion of **ISM042-2-004-2** (0.50 g, 3.38 mmol, 365 µL, 1.0 equiv.). The mixture was stirred at r.t. under N_2 atmosphere for 16 hrs. The reaction was diluted with water (20 mL) and extracted with EtOAc (20 mL × 2). The combined organic layers were washed with brine (30 mL), dried over anhydrous sodium sulfate, filtered and concentrated to afford **ISM042-2-004-3** (640 mg, 90.4% yield) as a colorless oil. ^1H NMR (400 MHz, $CDCl_3$) δ 8.23 (d, J = 5.60 Hz, 1H), 7.38 (s, 1H), 7.34 (s, 1H), 6.91 (d, J = 2.40 Hz, 1H), 6.88-6.86 (m, 1H), 3.93 (s, 3H). LCMS $[M+H]^+$: 210.1.

To a solution of **ISM042-2-004-3** (300 mg, 1.43 mmol, 1.0 equiv.) in THF (4 mL) were added 1-methylcyclopropanamine (308 mg, 2.86 mmol, 1.0 equiv.), $Pd_2(dba)_3$ (131 mg, 143 µmol, 0.1 equiv.), BINAP (178 mg, 286 µmol, 0.2 equiv.) and *t*BuONa (550 mg, 5.72 mmol, 4.0 equiv.). The mixture was stirred at 60 °C under N_2 atmosphere for 5 hrs. The reaction mixture was filtered and concentrated to afford a residue, which was purified by prep-HPLC to afford **ISM042-2-004** (60.0 mg, 238 µmol, 16.7% yield) as a white solid. ^1H NMR (400 MHz, CD_3OD) δ 7.83-7.76 (m, 1H), 7.63 (s, 1H), 7.40 (d, J = 0.40 Hz, 1H), 6.28-6.26 (m, 2H), 3.89 (s, 3H), 1.31 (s, 3H), 0.74-0.71 (m, 2H), 0.65-0.62 (m, 2H). LCMS $[M+H]^+$: 245.1.

ISM042-2-007

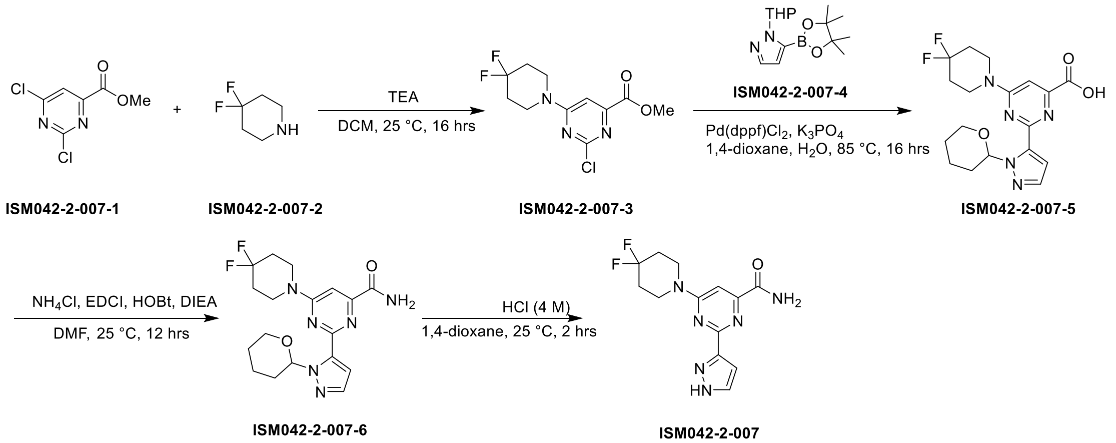

To a solution of **ISM042-007-1** (500 mg, 2.42 mmol, 1.0 equiv.) in DCM (2.5 mL) were added TEA (403 µL, 2.90 mmol, 1.2 equiv.) and **ISM042-007-2** (293 mg, 2.42 mmol, 1.0 equiv.). The mixture was stirred at r.t. for 16 hrs. The reaction mixture was diluted with water and extracted with EtOAc (3 mL × 3). The combined organic layers were dried over anhydrous sodium sulfate, filtered and concentrated. The residue was purified by silica gel chromatography (PE/EtOAc = 5/1) to afford **ISM042-007-3** (425 mg, 1.46 mmol, 60.33% yield) as a white solid. ^{1}H NMR (400 MHz, $CDCl_3$) δ 7.27 (s, 1H), 3.98 (s, 3H), 3.89 (br s, 4H), 2.14 - 2.02 (m, 4H). LCMS $[M+H]^+$: 292.1.

To a solution of **ISM042-007-3** (50 mg, 171 µmol, 1.0 equiv.) in 1,4-dioxane (3 mL) and H_2O (0.5 mL) were added **ISM042-007-4** (57.2 mg, 206 µmol, 1.2 equiv.), K_3PO_4 (109 mg, 514 µmol, 3.0 equiv.) and Pd(dppf)Cl_2 (12.5 mg, 17.1 µmol, 0.1 equiv.) under N_2 atmosphere. The mixture was stirred at 85 °C for 16 hrs. The reaction mixture was diluted with water and extracted with EtOAc (3 mL × 3). The combined organic layers were dried over anhydrous sodium sulfate, filtered and concentrated to afford **ISM042-007-5** (113 mg, crude) as a black brown oil. LCMS$[M+H]^+$: 393.9.

To a solution of **ISM042-007-5** (120 mg, 305 µmol, 1.0 equiv.) and NH_4Cl (33 mg, 611 µmol, 2.0 equiv.) in DMF (10 mL) were added EDCI (87.7 mg, 458 µmol, 1.5 equiv.), HOBT (61.8 mg, 458 µmol, 1.5 equiv.) and DIEA (197 mg, 1.53 mmol, 266 µL, 5.0 equiv.). The mixture was stirred at 25 °C for 12 hrs. The reaction mixture was diluted with water (20 mL) and extracted with EtOH (10 mL × 3). The combined organic layers were washed with brine (15 mL × 2), dried over anhydrous sodium sulfate, filtered and concentrated to afford **ISM042-007-6** (153 mg, crude) as a yellow oil. LCMS $[M+H]^+$: 393.2.

To a solution of **ISM042-007-6** (300 mg, 765 µmol, 1.0 equiv.) in 1,4-dioxane (3 mL) was added HCl (aq., 4 M, 1 mL, 5.23 equiv.). The mixture was stirred at r.t. for 2 hrs. The reaction mixture was concentrated and purified by prep-HPLC to afford **ISM042-2-007** (50 mg, 20.86% yield) as a white solid. ^{1}H NMR (400 MHz, CD_3OD) δ 7.67 (brs, 1H), 7.38 (s, 1H), 7.04 (s, 1H), 3.99 (brs, 4H), 2.16 - 2.04 (m, 4H). LCMS $[M+H]^+$: 309.2.

ISM042-2-009

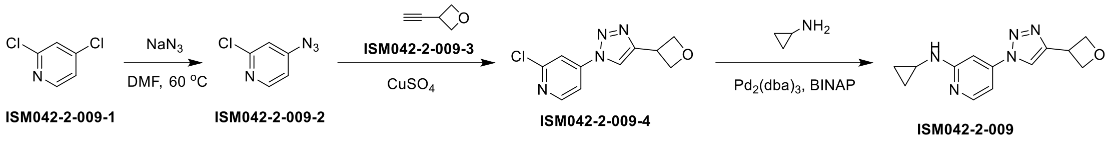

A mixture of **ISM042-2-009-1** (3.0 g, 12.53 mmol, 1.0 equiv.) and NaN_3 (977 mg, 15.04 mmol, 1.2 equiv.) in DMF (15 mL) was stirred at 60 °C for 36 hr under N_2 atmosphere. The reaction mixture was cooled to r.t. and poured into water (10 mL) and extracted with DCM (10 mL × 3). The water layers was poured into NaClO (aq.) for 12 hr and then discarded. The combined organic layers were dried over anhydrous sodium sulfate, filtered and concentrated to afford a residue, which was purified by silica gel chromatography (PE/EtOAc = 50/1) to afford **ISM042-2-009-2** (480 mg, 24.79% yield) as a white solid.

To a solution of **ISM042-2-009-2** (100 mg, 647.0 µmol, 1.0 equiv.) in THF (4 mL) and H_2O (1 mL) were added $CuSO_4$ (2.6 mg, 16.2 µmol, 2.5 µL, 0.025 equiv.), sodium ascorbate (6.4 mg, 32.4 µmol, 0.05 equiv.) and **ISM042-2-009-3** (80 mg, 970.5 µmol, 1.5 eq) in MeOH (6 mL). The mixture was stirred at r.t. for 3 hrs. The reaction mixture was extracted with EtOAc (5 mL × 3). The combined organic layers were dried over anhydrous sodium sulfate, filtered and concentrated to afford **ISM042-2-009-4** (170 mg, crude) as a yellow solid. LCMS $[M+H]^+$: 237.1.

To a solution of **ISM042-2-009-4** (140 mg, 591.6 µmol, 1.0 equiv.) in THF (1.4 mL) were added cyclopropanamine (82 µL, 1.18 mmol, 2.0 equiv.), ^{t}BuONa (114 mg, 1.18 mmol, 2.0 equiv.), $Pd_2(dba)_3$ (27.09 mg, 29.58 µmol, 0.5 equiv.) and BINAP (37 mg, 59.16 µmol, 0.1 equiv.). The mixture was stirred at 50 °C under N_2 atmosphere for 3.5 hrs. The reaction mixture was diluted with water (2 mL), and then extracted with EtOAc (5 mL × 2). The combined organic layers were dried over anhydrous sodium sulfate, filtered and concentrated. The obtained residue was purified by prep-HPLC to afford **ISM042-2-009** (35 mg, 23.0% yield) as a white solid. ^{1}HNMR (400 MHz, CD_3OD) δ 8.65 (s, 1H), 8.13 (d, *J* = 5.75 Hz, 1H), 7.21 (d, *J* = 1.63 Hz, 1H), 7.12 (dd, *J* = 5.63, 1.88 Hz, 1H), 5.08 (dd, *J* = 8.50, 5.88 Hz, 2H), 4.92 (t, *J* = 6.44 Hz, 2H), 4.52 (s, 1H), 2.61 (dt, *J* = 6.85, 3.27 Hz, 1H), 0.89 - 0.82 (m, 2H), 0.60 - 0.53 (m, 2H). LCMS $[M+H]^+$: 258.2.

ISM042-2-015

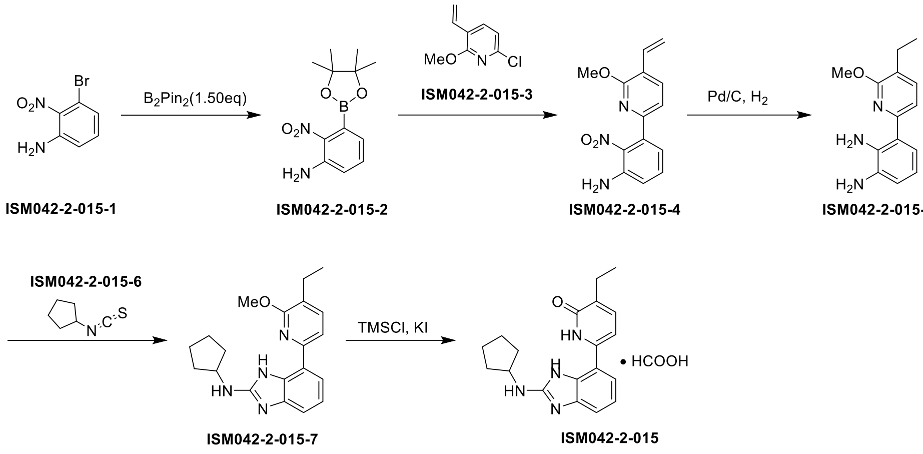

To a solution of **ISM042-2-015-1** (750 mg, 3.46 mmol, 1.0 equiv.) in 1,4-dioxane were added 4,4,5,5-tetramethyl-2-(4,4,5,5-tetramethyl-1,3,2-dioxaborolan-2-yl)-1,3,2-dioxaborolane (1.32 g, 5.18 mmol, 1.5 equiv.), Pd (dppf)Cl_2 (126 mg, 173 μmol, 0.5 equiv.) and KOAc (1.02 g, 10.4 mmol, 3.0 equiv.). The mixture was stirred at 85 °C under N_2 atmosphere for 12 hrs. The reaction mixture was filtered and the fliltrate was diluted with water (30 mL) and extracted with EtOAc (35 mL × 3). The combined organic layers were washed with brine (50 mL × 2), dried over anhydrous sodium sulfate, filtered and concentrated to afford **ISM042-2-015-2** (2.03 g, crude) as a yellow solid. LCMS $[M+H]^+$: 264.9.

To a solution of **ISM042-2-015-2** (900 mg, 3.41 mmol, 1.0 equiv.) in dioxane (15 mL) and H_2O (5 mL) were added **ISM042-2-014-3** (405 mg, 2.39 mmol, 0.70 equiv.), K_3PO_4 (2.17 g, 10.22 mmol, 3.0 equiv.) and Pd(dtbf)Cl_2 (222 mg, 341 μmol, 0.1 equiv.). The mixture was stirred at 100 °C under N_2 atmosphere for 12 hrs. The mixture was diluted with water (20 mL) then extracted with EtOAc (20 mL × 3). The combined organic layers were dried over anhydrous sodium sulfate and filtered. The filtrate was concentrated and purified by silica gel chromatography (PE/EtOAc = 5/1) to afford **ISM042-2-015-4** (420 mg, 45.43% yield) as a yellow solid. ^{1}HNMR (400 MHz, DMSO-d_6) δ 7.97 (d, J = 7.6 Hz, 1H), 7.24 - 7.36 (m, 2H), 6.99 (dd, J = 8.4, 1.2 Hz, 1 H), 6.77 - 6.87 (m, 2H), 6.11 (s, 2H), 5.96 (dd, J = 17.6, 1.2 Hz, 1H), 5.40 (dd, J = 11.2, 1.2 Hz, 1 H), 3.79 (s, 3H). LCMS $[M+H]^+$: 272.1.

To a solution of **ISM042-2-015-4** (420 mg, 1.55 mmol, 1.0 equiv.) in MeOH (4 mL) was added Pd/C (50.0 mg, 10% purity, 1.0 equiv.) under N_2 atmosphere. The suspension was degassed and purged with H_2 for 3 times. The mixture was stirred under H_2 (15 Psi.) at r.t. for 12 hrs. The mixture was filtered and concentrated to afford **ISM042-2-015-5** (320 mg, 1.32 mmol, 84.9% yield) as a white solid. ^{1}HNMR (400 MHz, DMSO-d_6) δ 7.60 (d, J = 7.6 Hz, 1H), 7.20 (d, J = 7.6 Hz, 1H), 6.80 (d, J = 7.6 Hz, 1H), 6.57 - 6.62 (m, 1H), 6.44 - 6.51 (m, 1H), 5.68 (s, 2H), 4.58 (s, 2H), 3.92 (s, 3H), 2.57 (q, J = 7.6 Hz, 2H), 1.17 (t, J = 7.6 Hz, 3H). LCMS $[M+H]^+$: 244.0.

To a solution of **ISM042-2-015-5** (300 mg, 1.23 mmol, 1.00 equiv.) in CH_3CN (15 mL) were added **ISM042-2-015-6** (165 mg, 1.29 mmol, 1.05 equiv.) and DIPEA (286 μL, 1.85 mmol, 1.5 equiv.). The mixture was stirred at 80 °C for 10 hrs. The reaction mixture was concentrated to afford **ISM042-2-015-7** (400 mg, crude) as a white solid. LCMS $[M+H]^+$: 337.1.

To a solution of **ISM042-2-015-7** (380 mg, 1.13 mmol, 1.0 equiv.) in CH_3CN (12 mL) were added KI (563 mg, 3.39 mmol, 3.0 equiv.) and TMSCl (430 μL, 3.39 mmol, 3.0 equiv.). The mixture was stirred at 70 °C for 28 hrs. The reaction is cooled to rt, diluted with water (20 mL) and extracted with DCM (30 mL× 3). The combined organic layer was washed with brine (30 mL) , dried over anhydrous sodium sulfate, filtered

and concentrated. The obtained residue was purified by prep-HPLC to afford **ISM042-2-015** (230 mg, 74.4% yield) as a yellow solid. ^{1}HNMR (400 MHz, CD_3OD) δ 8.12 (s, 1H), 7.54 (d, J = 6.0 Hz, 1H), 7.52 (d, J = 5.9 Hz, 1H), 7.27 (d, J = 7.6 Hz, 1H), 7.11 (t, J = 7.9 Hz, 1H), 6.99 (d, J = 7.2 Hz, 1H), 4.21 (m, 1H), 2.59 (q, J = 7.6 Hz, 2H), 2.10 - 2.21 (m, 2H), 1.77 - 1.88 (m, 2H), 1.61 - 1.77 (m, 4H), 1.23 (t, J = 7.6 Hz, 3H). LCMS $[M+H]^+$: 323.1.

ISM042-2-019

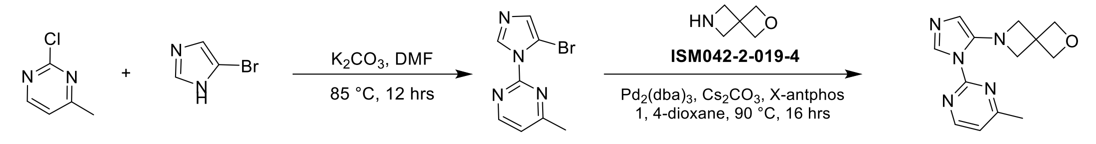

ISM042-2-019-1 **ISM042-2-019-2** **ISM042-2-019-3** **ISM042-2-019**

To a solution of **ISM042-2-019-1** (1.0 g, 7.78 mmol, 1.0 equiv.) and **ISM042-2-019-2** (1.26 g, 8.56 mmol, 1.1 equiv.) in DMF (20 mL) was added K_2CO_3 (2.15 g, 15.56 mmol, 2.0 equiv.). The mixture was stirred at 85 °C for 12 hrs. The reaction mixture was diluted with water (40 mL) and extracted with EtOAc (30 mL × 3). The combined organic layers were washed with brine (50 mL × 2), dried over anhydrous sodium sulfate and filtered. The filtrate was concentrated to afford **ISM042-2-019-3** (1.68 g, 90.34% yield) as a light yellow solid. ^{1}H NMR (400 MHz, CD_3OD) δ 8.61 (d, J = 5.2 Hz, 1H), 8.57 (d, J = 1.2 Hz, 1H), 7.99 (d, J = 1.6 Hz, 1H), 7.30 (d, J = 5.2 Hz, 1H), 2.58 (s, 3H). LCMS $[M+H]^+$: 239.1.

To a solution of **ISM042-2-019-3** (300 mg, 1.25 mmol, 1.0 equiv.) in 1,4-dioxane (20 mL) were added **ISM042-2-019-4** (124 mg, 1.25 mmol, 1.0 equiv.), Xantphos (145 mg, 251 μmol, 0.2 equiv.), Cs_2CO_3 (1.23 g, 3.76 mmol, 3.0 equiv.) and $Pd_2(dba)_3$ (115 mg, 125 μmol, 0.1 equiv.). The mixture was stirred at 100 °C under N_2 atmosphere for 16 hrs. The reaction mixture was diluted with water and extracted with EtOAc (10 mL × 3). The combined organic layers were washed with brine (15 mL × 2), dried over anhydrous sodium sulfate and filtered. The filtrate was concentrated and purified by prep-HPLC to afford **ISM042-2-019** (218 mg, 62.20% yield) as a white solid. ^{1}H NMR (400 MHz, CD_3OD) δ 8.55 (d, J = 5.2 Hz, 1 H), 8.39 (s, 1H), 7.21 (d, J = 4.8 Hz, 1H), 7.11 (s, 1H), 4.87 (s, 4H), 3.98 (s, 4H), 2.55 (s, 3H). LCMS $[M+H]^+$**:** 258.2.

ISM042-2-025

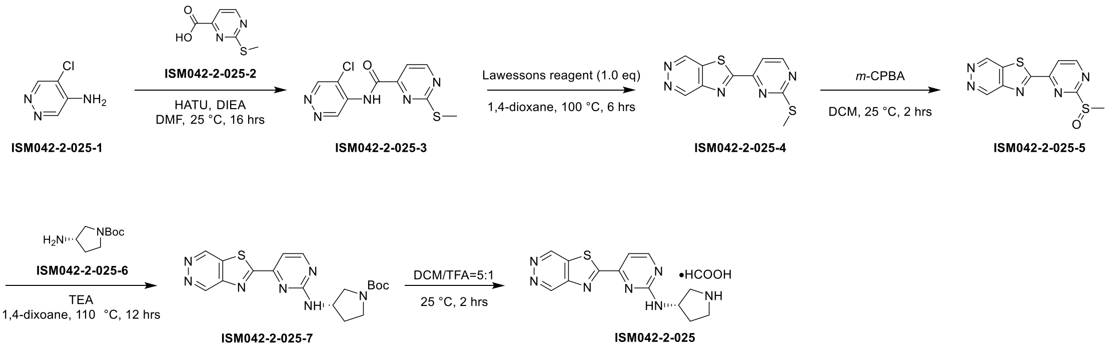

To a solution of **ISM042-2-025-1** (1.86 g, 14.4 mmol, 1.0 equiv.) and **ISM042-2-025-2** (2.93 g, 17.2 mmol, 1.2 equiv.) in DMF (60 mL) were added DIPEA (5.57 g, 43.1 mmol, 7.50 mL, 3.0 equiv.) and HATU (8.19 g, 21.5 mmol, 1.5 equiv.). The mixture was stirred at r.t. for 16 hrs. The reaction mixture was diluted with water (6 mL) and extracted with EtOAc (5 mL × 3). The combined organic layers were washed with brine (8 mL × 2), dried over anhydrous sodium sulfate and filtered. The filtrate was concentrated and triturated with CH_3CN at r.t. for 30 min. The mixture was filtered to afford **ISM042-2-025-3** (3.80 g, 93.95% yield) as a yellow solid.

To a solution of **ISM042-2-025-3** (1.50 g, 5.32 mmol, 1.0 equiv.) in 1,4-dioxane (40 mL) was added Lawesson's reagent (2.15 g, 5.32 mmol, 1.0 equiv.). The mixture was stirred at 100 °C for 6 hrs. The reaction was quenched by water (25 mL) and treated with sodium hydroxide (aq. 20%wt) to remove the unreacted Lawesson's reagent. The mixture extracted with EtOAc (35 mL × 3). The combined organic layers were washed with brine (50 mL × 2), dried over anhydrous sodium sulfate and filtered. The filtrate was concentrated and triturated with CH_3CN at r.t. for 30 min. The mixture was filtered to afford **ISM042-2-025-4** (1.25 g, 89.84% yield) as a brown solid. ^{1}H NMR (400 MHz, CD_3OD) δ 10.13 (d, J = 1.2 Hz, 1H), 9.97 (d, J = 1.6 Hz, 1H), 8.94 (d, J = 5.2 Hz, 1H), 8.01 (d, J = 5.2 Hz, 1H), 2.63 (s, 3H). LCMS $[M+H]^+$: 262.1;

To a solution of **ISM042-2-025-4** (1.20 g, 4.59 mmol, 1.0 equiv.) in DCM (20 mL) was added *m*-CPBA (60 wt%, 3.30 g, 11.5 mmol, 2.5 equiv.). The mixture was stirred at 25 °C for 2 hrs. The mixture was stirred at 100 °C for 6 hrs. The reaction mixture was quenched with Na_2SO_3 (aq., 15 mL) at 0 °C and then extracted with DCM (20 mL × 3). The combined organic layers were washed with brine (35 mL × 2), dried over anhydrous sodium sulfate, filtered and concentrated to afford **ISM042-2-025-5** (1.23 g, crude) as a yellow solid. LCMS $[M+H]^+$: 277.9.

To a solution of **ISM042-2-025-5** (800 mg, 2.88 mmol, 1.0 equiv.) and **ISM042-2-025-6** (2.69 g, 14.4 mmol, 5.0 equiv.) in 1,4-dioxane (15 mL) was added TEA (1.46 g, 14.4 mmol, 2.0 mL, 5.0 equiv.). The mixture was stirred at 110 °C for 12 hrs. The reaction mixture was quenched by water (10 mL), and then extracted with EtOAc (10 mL × 3). The combined organic layers were washed with brine (15 mL × 2), dried over anhydrous sodium sulfate and filtered. The filtrate was concentrated and purified by prep-HPLC to afford **ISM042-2-025-7** (800 mg, crude) as a yellow solid. LCMS $[M+H]^+$: 400.2.

To a solution of **ISM042-2-025-7** (100 mg, 250 μmol, 1.0 equiv.) in DCM (1 mL) was added TFA (185 μL, 2.50 mmol, 10.0 equiv.). The mixture was stirred at 25 °C for 2 hrs. The reaction mixture was concentrated, diluted with water (3 mL) and neutealized by $NaHCO_3$ (aq.). The mixtrure was extracted with a mixture of DCM and ^{i}PrOH (8:1, 5 mL × 5). The combined organic layers were dried over anhydrous sodium sulfate and filtered. The filtrate was concentrated and purified by prep-HPLC to afford **ISM042-2-025** (100 mg, 66.35% yield) as a yellow solid. ^{1}H NMR: (400 MHz, CD_3OD) δ 9.95 (d, J =

1.2 Hz, 1H), 9.82 (d, *J* = 1.2 Hz, 1H), 8.63 (d, *J* = 4.8 Hz, 1H), 8.54 (s, 1H), 7.63 (d, *J* = 4.8 Hz, 1H), 4.60 - 4.72 (m, 1H), 3.49 - 3.69 (m, 2H), 3.40 - 3.49 (m, 2H), 2.47 - 2.42 (m, 1H), 2.17 - 2.28 (m, 1H). LCMS $[M+H]^+$: 300.2.

ISM042-2-048

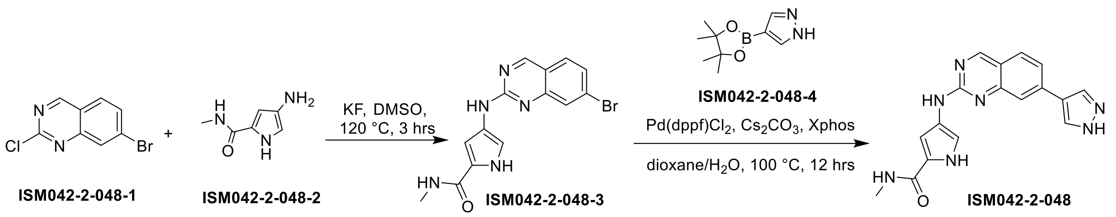

A mixture of **ISM042-2-048-1** (244 mg, 1.01 mmol, 1.0 equiv.) and **ISM042-2-048-2** (140 mg, 1.01 mmol, 1.0 equiv.) in DMSO (5 mL) was stirred at 25 °C for 1 hr, followed by the addition of KF (88 mg, 1.51 mmol, 1.5 equiv.). The resulting mixture was stirred at 120 °C for 2 hrs. The mixture was cooled to r.t. and was poured into water (10 mL). The mixture was placed in ultrasound bath for 10 min, and then filtered to afford **ISM042-2-048-3** (350 mg, 77.28% yield) as a yellow solid. LCMS $[M+H]^+$: 346.0.

To a solution of **ISM042-2-048-3** (130 mg, 288 µmol, 1.0 equiv.) in dioxane (5 mL) and H_2O (1 mL) were added **ISM042-2-048-4** (112 mg, 577 µmol, 2.0 equiv.), Xphos (28 mg, 57.7 µmol, 0.2 equiv.), Pd(dppf)Cl_2 (21 mg, 28.8 µmol, 0.1 equiv.) and Cs_2CO_3 (282 mg, 866 µmol, 3.0 equiv.). The mixture was stirred at 100 °C under N_2 atmosphere for 12 hrs. The reaction mixture was diluted with EtOAc (5 mL), and then filtered. The filtrate was concentrated and purified by prep-HPLC to afford **ISM042-2-048** (17.0 mg, 17.6% yield) as a yellow solid. ^{1}H NMR (400 MHz, DMSO-d_6) δ 13.10 (s, 1H), 11.13 (s, 1H), 9.58 (s, 1H), 9.07 (s, 1H), 8.44 (s, 1H), 8.15 (s, 1H), 7.96 (q, J = 4.2 Hz, 1H), 7.79 (d, J = 8.5 Hz, 1H), 7.77 (s, 1H), 7.58 (dd, J = 8.3, 1.7 Hz, 1H), 7.50 (s, 1H), 6.85 (s, 1H), 2.74 (d, J = 4.5 Hz, 3H). LCMS $[M+H]^+$: 334.2.

ISM042-2-049

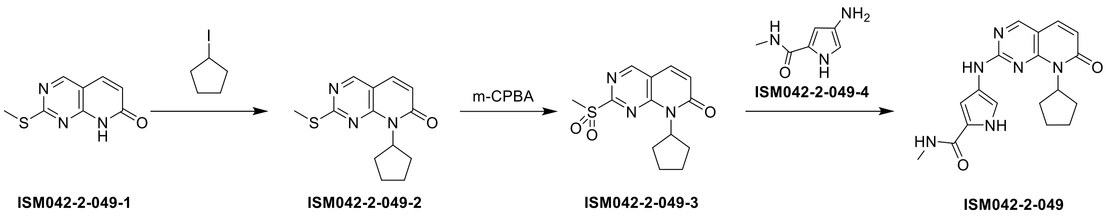

To a suspension of K_2CO_3 (214 mg, 1.55 mmol, 3.0 equiv.) in DMF (4 mL) was added **ISM042-2-049-1** (100 mg, 517 µmol, 1.0 equiv.). The reaction mixture was heated to 50 °C, resulting in a brown solution. The solution was cooled to rt, followed by the addition of iodocyclopentane (119 µL, 1.04 mmol, 2.0 equiv.). The reaction was stirred at 70 °C for 2 hrs. The reaction mixture was diluted with water (50 mL) and extracted with EtOAc (80 mL × 2). The combined organic layers were washed with brine (20 mL × 2), dried over anhydrous sodium sulfate, and filtered. The filtrate was concentrated and purified by silica gel chromatography (PE/EtOAc = 2/1) to afford **ISM042-2-049-2** (50.0 mg, 36.9 % yield) as yellow solid. LCMS $[M+H]^+$: 262.1.

To a solution of **ISM042-2-049-2** (60.0 mg, 229 µmol, 1.0 equiv.) in DCM (2 mL) was added *m*-CPBA (60 wt%, 132 mg, 459 µmol, 2.0 equiv.). The mixture was stirred at r.t. for 2 hrs. The reaction mixture was diluted with water (50 mL) and extracted with EtOAc (80ml × 2). The combined organic layers were washed with brine (20 mL × 2), dried over anhydrous sodium sulfate, filtered, and concentrated to afford **ISM042-2-049-3** (50 mg, 74.2% yield) as yellow solid. LCMS $[M+H]^+$: 294.0.

To a solution of **ISM042-2-049-3** (40 mg, 136 µmol, 1.0 equiv.) and **ISM042-2-049-4** (95 mg, 681 µmol, 5.0 equiv.) in dioxane (5 mL) was added TEA (95 µL, 681 µmol, 5.0 equiv.). The mixture was stirred at 110°C for 2 hrs. The reaction mixture was diluted with water (50 mL) and extracted with EtOAc (80 mL × 2). The combined organic layers were washed with brine (20 mL × 2), dried over anhydrous sodium sulfate, and filtered. The filtrate was concentrated and purified by prep-HPLC to afford **ISM042-2-049** (3.0 mg, 6.18% yield) as a yellow solid. ^{1}H NMR (400 MHz, CD_3OD) δ 8.61 (s, 1H), 7.71 (d, J = 9.3 Hz, 1H), 7.28 (d, J = 1.7 Hz, 1H), 6.80 (s, 1H), 6.33 (d, J = 9.3 Hz, 1H), 6.09 – 5.95 (m, 1H), 2.90 (s, 3H), 2.33 (td, J = 10.1, 8.8, 4.4 Hz, 2H), 2.08 – 1.93 (m, 2H), 1.95 – 1.83 (m, 2H), 1.75 – 1.63 (m, 2H). LCMS $[M+H]^+$: 353.2.

ISM042-2-050

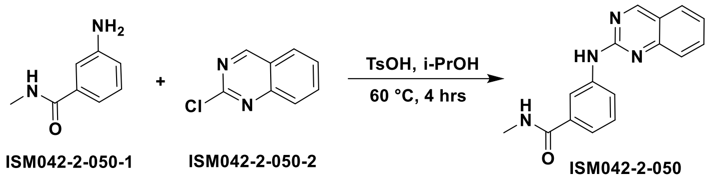

To a solution of **ISM042-2-050-1** (100 mg, 665 µmol, 1.0 equiv.) in ^{i}PrOH (2 mL) were added **ISM042-2-050-2** (131 mg, 799 µmol, 1.2 equiv.) and TsOH (229 mg, 1.33 mmol, 2.0 equiv.). The mixture was stirred at 60°C for 4 hrs. The reaction mixture was concentrated and purified by prep-HPLC to afford **ISM042-2-050** (50 mg, 26.17% yield) as a white solid. ^{1}H NMR (400 MHz, CD_3OD) δ ppm 9.19 (s, 1H), 8.32 - 8.40 (m, 1H), 8.02 - 8.14 (m, 1H), 7.69 - 7.91 (m, 3H), 7.32 - 7.53 (m, 3H), 2.95 (s, 3H). LCMS $[M+H]^+$: 279.1.

ISM042-2-052

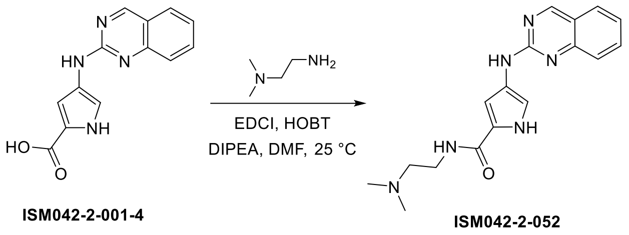

To a solution of **ISM042-2-001-4** (100 mg, 393 µmol, 1.0 equiv.) in DMF (1 mL) were added N',N'-dimethylethane-1,2-diamine (45 µL, 412.9 µmol, 1.0 equiv.), HOBT (58.46 mg, 432.66 µmol, 1.1 equiv.), EDCI (83 mg, 432.66 µmol, 1.1 equiv.) and DIPEA (137 µL, 786.65 µmol, 2.0 equiv.). The mixture was stirred at r.t. under N_2 atmosphere for 16 hrs. The reaction mixture was diluted with water (2 mL) and extracted with EtOAc (3 mL × 3). The combined organic layers were washed with brine (5 mL × 2), dried over anhydrous sodium sulfate and filtered. The filtrate was concentrated and purified by prep-HPLC to afford **ISM042-2-052** (15.2 mg, 11.73% yield) as a yellow solid. ^{1}H NMR (400 MHz, CD_3OD) δ ppm 9.07 (s, 1H), 7.79 (d, J = 8.0 Hz, 1H), 7.73 (t, J = 8.2 Hz, 1H), 7.64 (d, J = 8.5 Hz, 1H), 7.52 (s, 1H), 7.29 (t, J = 7.4 Hz, 1H), 6.96 (d, J = 1.6 Hz, 1H), 3.50 (t, J = 6.8 Hz, 2H), 2.57 (t, J = 6.9 Hz, 2H), 2.32 (s, 6H). LCMS $[M+H]^+$: 325.0.

ISM042-2-053

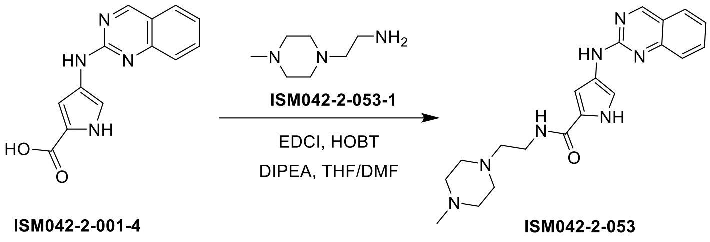

To a solution of **ISM042-2-001-4** (100 mg, 393.3 μmol, 1.0 equiv.) in DMF (1 mL) were added **ISM042-2-053-1** (59 mg, 412.9 μmol, 1.05 equiv.), EDCI (83 mg, 432.66 μmol, 1.1 equiv.), DIPEA (137 μL, 786.65 μmol, 2.0 equiv.) and HOBT (58 mg, 432.66 μmol, 1.1 equiv.). The mixture was stirred at r.t. under N_2 atmosphere for 3 hrs. The reaction mixture was concentrated and purified by prep-HPLC to afford **ISM042-2-053** (18.7 mg, 12.05% yield) as a yellow solid. ^{1}H NMR (400 MHz, CD_3OD) δ ppm 9.08 (s, 1H), 7.79 (d, *J* = 8.0 Hz, 1H), 7.73 (ddd, *J* = 8.5, 6.8, 1.5 Hz, 1H), 7.64 (d, *J* = 8.5 Hz, 1H), 7.51 (s, 1H), 7.30 (d, *J* = 7.4 Hz, 1H), 6.95 (d, *J* = 1.6 Hz, 1H), 3.50 (d, *J* = 6.8 Hz, 2H), 2.69 – 2.43 (m, 10H), 2.29 (s, 3H). LCMS $[M+H]^+$: 380.3.

ISM042-2-055

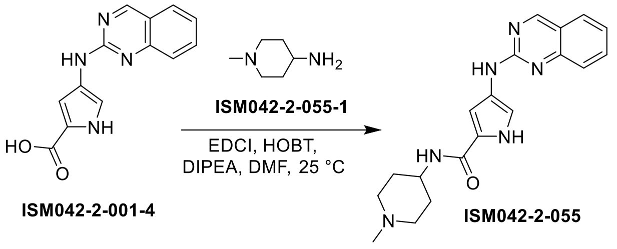

To a solution of **ISM042-2-001-4** (100 mg, 393 µmol, 1.0 equiv.) in DMF (1.0 mL) were added **ISM042-2-055-1** (59 mg, 412.99 µmol, 1.0 equiv.), EDCI (83 mg, 432 µmol, 1.1 equiv.), DIPEA (137 uL, 786 µmol, 2.0 equiv.) and HOBT (58 mg, 432 µmol, 1.1 equiv.). The mixture was stirred at r.t. under N_2 atmosphere for 3 hrs. The reaction mixture was concentrated and purified by prep-HPLC to afford **ISM042-2-055** (33.0 mg, 6.78% yield) as a yellow solid. ^{1}H NMR (400 MHz, CD_3OD) δ ppm 9.09 (s, 1H), 7.81 (d, *J* = 8.0 Hz, 1H), 7.75 (ddd, *J* = 8.4, 6.9, 1.5 Hz, 1H), 7.66 (d, *J* = 8.5 Hz, 1H), 7.54 (s, 1H), 7.31 (ddd, *J* = 8.0, 6.9, 1.2 Hz, 1H), 7.01 (d, *J* = 1.6 Hz, 1H), 3.93 - 3.81 (m, 1H), 2.94 (d, *J* = 11.9 Hz, 2H), 2.33 (s, 3H), 2.19 (t, *J* = 11.9 Hz, 2H), 1.97 (d, *J* = 12.5 Hz, 2H), 1.69 (qd, *J* = 12.2, 3.8 Hz, 2H). LCMS $[M+H]^+$: 351.2.
